# A novel endobronchial approach to massive hemoptysis complicating silicone Y-stent placement for tracheobronchomalacia: A case report

**DOI:** 10.1097/MD.0000000000009980

**Published:** 2018-02-23

**Authors:** Changwan Ryu, Daniel Boffa, Kyle Bramley, Margaret Pisani, Jonathan Puchalski

**Affiliations:** a Yale School of Medicine, Department of Internal Medicine, Section of Pulmonary, Critical Care, and Sleep Medicine; b Yale School of Medicine, Department of Thoracic Surgery, New Haven, CT, USA.

**Keywords:** biocompatible surgical sealant, oxidized regenerated cellulose, silicone Y-stent, tracheobronchomalacia

## Abstract

**Rationale:**

Airway stabilization for severe, symptomatic tracheobronchomalacia (TBM) may be accomplished by silicone Y-stent placement. Common complications of the Y-stent include mucus plugging and granulation tissue formation.

**Patient concerns:**

We describe a rare case of massive hemoptysis originating from a silicone Y-stent placed for TBM.

**Diagnoses:**

An emergent bronchoscopy showed an actively bleeding, pulsatile vessel at the distal end of the left bronchial limb of the Y-stent. It was felt that the bleeding was caused by, or at least impacted by, the distal left bronchial limb of the Y-stent eroding into the airway wall.

**Interventions:**

We hypothesized that placement of oxidized regenerated cellulose (ORC) would provide initial hemostasis, and the subsequent placement of a biocompatible surgical sealant would lead to definitive resolution.

**Outcomes:**

ORC provided sufficient hemostasis and the subsequent synthetic polymer reinforced the tissue for complete cessation of the bleed.

**Lessons:**

The combined use of ORC and a biocompatible surgical sealant provided long-term management for life-threatening hemoptysis, and potentially morbid procedures such as embolization or surgery were avoided by advanced endobronchial therapy.

## 1 Introduction

Tracheobronchomalacia (TBM) is an anomaly of the central airways characterized by the excessive expiratory collapsibility of the tracheal and bronchial walls.^[1,2]^ Congenital causes include prematurity and cartilage abnormalities, while acquired causes stem from prolonged intubation, severe obstructive lung disease, and recurrent tracheobronchitis.^[1,2]^ As a result of either etiology, there is atrophy of the longitudinal elastic fibers of the pars membranacea, an increase in membranous tracheal diameter, and fragmentation of cartilaginous rings, leading to weakness of the tracheobronchial wall and supporting cartilage.^[1]^ The most common symptoms include cough, dyspnea, and recurrent infection.^[1,3]^

**Figure 1:**
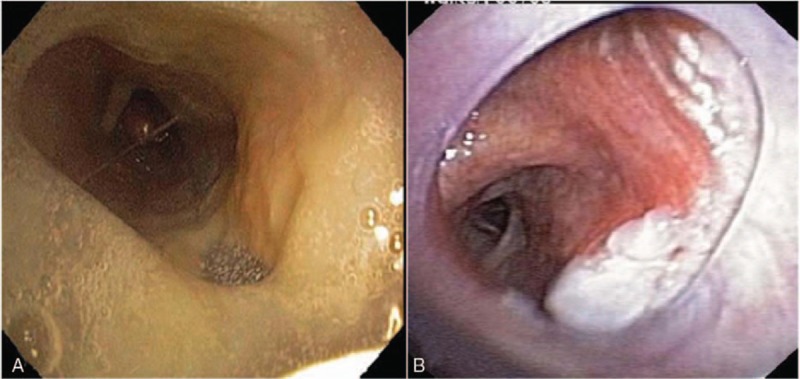
Complications of silicone Y-stent placement. Complications of silicone Y-stents typically occur within the first 3 months of placement, and the most common include (A) infectious biofilm formation and (B) granulation tissue formation, which may be significant.

Airway stabilization for severe, symptomatic TBM has been accomplished by silicone Y-stent placement^[3–5]^ with subsequent improvement in dyspnea, quality of life, and functional status.^[3]^ Not uncommonly, the Y-stent has led to the development of mucus plugging, biofilm formation, and granulation tissue development (Fig. 1A and B).^[3]^ We describe a rare, life-threatening case of massive hemoptysis complicating Y-stent placement and a novel therapeutic approach via the endobronchial application of oxidized regenerated cellulose (ORC) and a biocompatible surgical sealant.

## 2 Case presentation

A 65-year-old Caucasian woman with a past medical history significant for coronary artery disease requiring recent stenting, allergic asthma, chronic obstructive pulmonary disease (COPD), and severe TBM secondary to her obstructive lung diseases presented to our institution with massive hemoptysis. Three months prior to presentation she underwent placement of a silicone Y-stent as destination therapy for symptomatic TBM. At this point, she experienced recurrent hospitalizations for respiratory failure and refractory dyspnea despite maximization of other medical therapies, including airway clearance techniques, bronchodilators, physical therapy, and continuous positive airway pressure (CPAP). Tracheobronchoplasty was initially considered after Y-stent placement but was not viable because, in addition to declining surgical approaches to management, her multiple co-morbidities and recurrent hospitalizations severely limited her functional status. One month prior to presentation she underwent uneventful argon plasma coagulation to ablate granulation tissue. Upon arrival to our emergency department, she complained of progressive hemoptysis and worsening dyspnea for 2 hours. Physical examination revealed hypoxemic respiratory failure with hemoptysis of 250 ml of frank blood within an hour of presentation and diminished breath sounds in the left lung. She required intubation and bronchoscopy showed an actively bleeding, pulsatile vessel at the distal end of the left bronchial limb of the Y-stent. It was felt that the bleeding was caused by, or at least impacted by, the distal left bronchial limb of the Y-stent eroding into the airway wall. Given her critical condition, she was taken emergently to the operating room (OR) for removal of her stent. Interventional radiology was briefly considered, but given our impression that the stent needed to be removed and bronchoscopy was required, plus our ability to topically treat the area, embolization was reserved for bronchoscopic failure.

## 3 Airway management

Following her initial bronchoscopy, hemostasis was achieved via placement of an Arndt endobronchial blocker (Cook Critical Care, Bloomington, IN). In the time it took to bring her emergently to the OR, repeat bronchoscopy demonstrated bleeding cessation. The endobronchial blocker and Y-stent were then removed to allow definitive management in this “window of opportunity.” With this, however, bleeding was again evident and ORC (Surgicel, Ethicon, Somerville, NJ) was immediately placed at the bleeding site through the rigid bronchoscope, again leading to bleeding cessation. We felt a more definitive approach would be required given the ongoing bleeding at a localized source.

**Figure 2:**
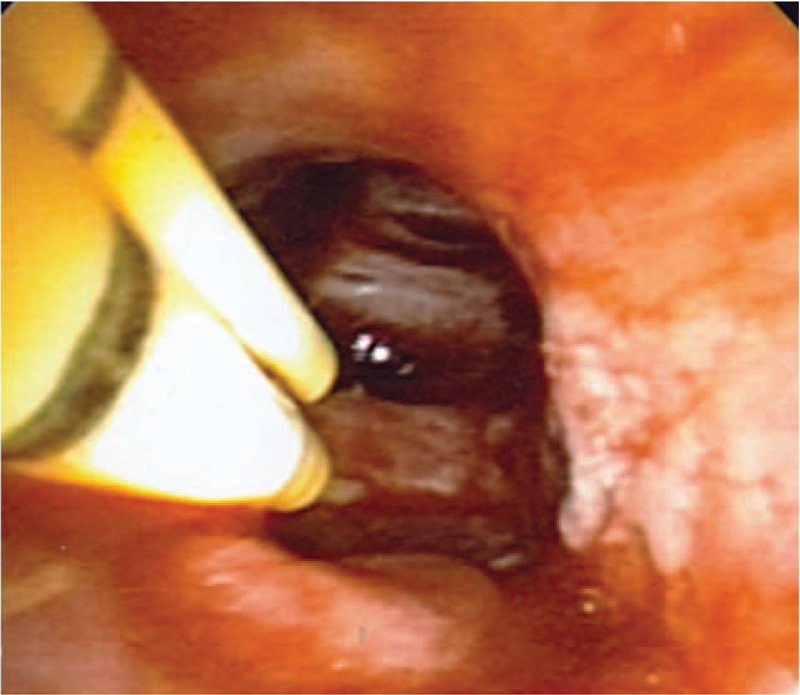
Application of the surgical sealant. Dual ureteral catheters were placed in distal left mainstem bronchus and used to administer each component of the sealant, enabling endobronchial mixing and permanent bleeding control.

**Figure 3:**
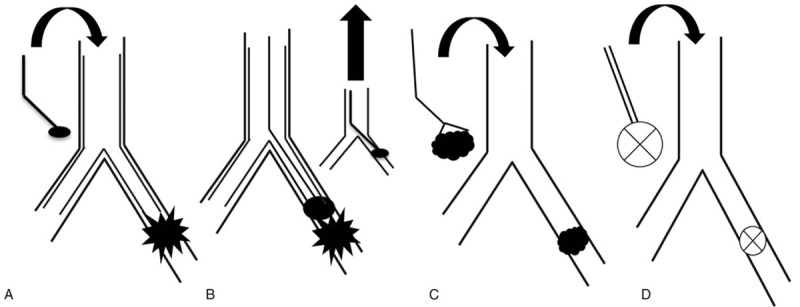
Schematic of airway management. (A) After visualization of pulsatile blood flow in distal left mainstem bronchus (starburst) at the termination of the Y stent bronchial limb, an endobronchial blocker (oval) was placed for temporary cessation of hemoptysis and to facilitate transfer to the operating room (OR). (B) In the OR, the endobronchial blocker and Y-stent were removed and initial hemostasis was achieved using (C) surgicel (cloud), placed endoscopically with forceps. Bleeding stopped and subsequently (D) dual ureteral catheters were used to administer each component of the sealant, enabling endobronchial mixing and permanent bleeding control. OR = operating room.

To this end, in collaboration with thoracic surgery, 2 ureteral catheters were tied in tandem using stitches to ensure they terminated equidistant in the airways. The 6 French size (Fr) polyurethane catheters (Bard, Covington, GA) were chosen for their length (70 cm), combined size (effectively 12 Fr), and conceptually that each of the 2 sealant components could be mixed together at the distant site of bleeding, thus preventing sealing of the delivery system during actual administration. The tied catheters were advanced through the rigid bronchoscope using direct visualization. Thereafter, the biocompatible surgical sealant (CoSeal, Baxter, Deerfield, IL) was administered endobronchially at the precise bleeding site using the two-syringe system attached to the catheters. Placement of these catheters distally in the left mainstem (Fig. 2) enabled endobronchial mixing of the sealant directly at the bleeding source. She was extubated within 24 hours and was successfully discharged home. A complete schematic of her airway management is shown in Figure 3.

**Figure 4:**
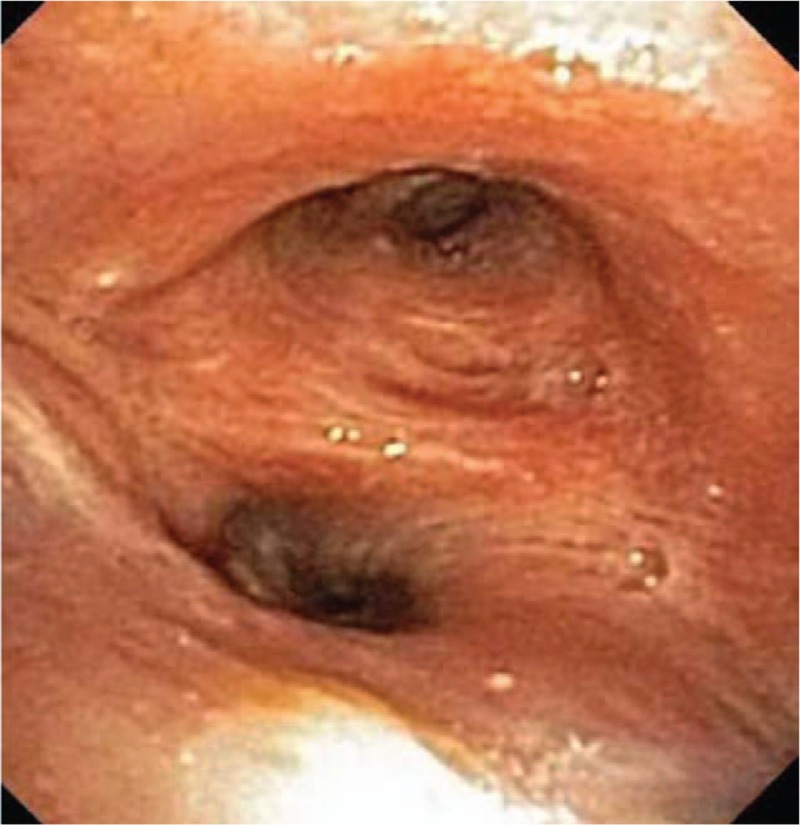
Follow-up bronchoscopy. Bronchoscopy 1 month later demonstrated no active bleeding and improvement of the granulation tissue.

A follow-up bronchoscopy 1 month later demonstrated mucosal healing (Fig. 4), and she reported no additional episodes of hemoptysis. In the 2 years following removal of her stent, there have been no new complications to report and her respiratory status has remained satisfactory despite removal of the stent.

## 4 Discussion

The use of silicone Y-stents for airway stabilization in severe TBM has demonstrated improvement in dyspnea, quality of life, and functional status.^[3]^ Complications typical occur within the first 3 months of stent placement and include obstruction from mucus plugging, stent migration, infection, and granulation tissue formation.^[3]^ These complications are typically treated with mucolytics, cough suppressants, suctioning, and antibiotics, only rarely necessitating stent removal.^[3,4]^ In the few reported cases of hemoptysis following silicone Y-stent placement, the proposed etiologies include local erosion from stent apposition, granulation tissue formation resulting in irritation, and fistula formation with adjacent blood vessels.^[6]^ In our case, given that the patient previously had granulation tissue that required ablation, we suspect she had ongoing irritation from the stent creating local erosion and prompting pulsatile bleeding from a bronchial artery.

The use of bronchoscopy to control massive hemoptysis commonly involves endobronchial instillation of iced saline^[7]^ or vasoconstrictive agents such as epinephrine,^[8]^ tranexamic acid,^[9]^ or fibrinogen–thrombin combinations,^[10]^ at the bleeding site. Advanced bronchoscopic procedures include argon plasma coagulation, electrocautery, and laser photocoagulation when a bleeding lesion can be identified.^[8]^ Bronchial artery embolization and surgery are reserved in cases where hemoptysis cannot be managed bronchoscopically.^[8]^

The individual use of ORC^[6]^ and biocompatible surgical sealants^[11]^ to achieve hemostasis in hemoptysis has been described, although this combination has not been previously reported. We hypothesized that ORC provided sufficient hemostasis, and the subsequent synthetic polymer reinforced the tissue for complete cessation of the bleed.

ORC contains caustic properties that react with blood to form an artificial coagulum that provides the substrate for further clotting.^[12]^ Its availability in multiple forms, such as an absorbable mesh, has led to its wide application to control bleeding in highly vascular sites.^[6,12]^ Its role in the management of hemoptysis via endobronchial application has been described,^[6]^ and its use in the treatment of hemoptysis remains an off-label indication.

Biocompatible surgical sealants achieve hemostasis by promoting coagulation and providing a mechanical barrier at the site of bleeding.^[13]^ They are frequently utilized during vascular surgery to control bleeding at anastomotic sites.^[13,14]^ While various sealants are available,^[13]^ we employed polyethylene glycol (PEG) polymers that form a highly adhesive hydrogel when mixed together.^[15]^ The hydrogel then binds to tissue surface proteins to achieve hemostasis with a rapid onset of action.^[15]^ In our case, we employed urethral catheters as a conduit to deliver each of the polymers because the normal admixing device is not plausible for airways. Although the use of PEG polymers to treat hemoptysis has yet to be described and is presently an off-labeled indication for its treatment, we believed PEG polymers to be appropriate for its application in the airways because it is more compliant than other available sealants.^[13,14]^ Given that the volume of application may increase following admixture, we were prepared to bronchoscopically remove any plug or airway occlusion. In this regard, the rigid bronchoscope was in place and additional balloons, forceps, and recovery and ablative tools were immediately available if required. We also employed the assistance of a thoracic surgeon (DB), who has had extensive experience with CoSeal and is thoroughly familiar with its properties, to help navigate any potential pitfalls in the novel application of this sealant. We advocate a similar approach for future cases.

## 5 Conclusion

Massive hemoptysis is a rare complication of silicone Y-stent placement. We present a novel method of managing this life-threatening complication with advanced endobronchial therapy that combined the placement of oxidized regenerated cellulose with a biocompatible surgical sealant. In addition to minimizing ongoing injury, we were able to provide a long-term treatment strategy that avoided potentially morbid procedures, such as embolization or surgery.

## References

[1] Carden KA Boiselle PM Waltz DA Tracheomalacia and tracheobronchomalacia in children and adults: an in-depth review. Chest 2005;127:984–1005. 15764786 10.1378/chest.127.3.984

[2] Septimiu D Murgu HGC Tracheobronchomalacia and excessive dynamic airway collapse. Respirology 2006;11:388–406. 16771908 10.1111/j.1440-1843.2006.00862.x

[3] Ernst A Majid A Feller-Kopman D Airway stabilization with silicone stents for treating adult tracheobronchomalacia: a prospective observational study. Chest 2007;132:609–16. 17699133 10.1378/chest.06-2708

[4] Oki M Saka H New dedicated bifurcated silicone stent placement for stenosis around the primary right carina. Chest 2013;144:450–5. 23471292 10.1378/chest.12-2834

[5] Makris D Marquette CH Tracheobronchial stenting and central airway replacement. Curr Opin Pulm Med 2007;13:278–83. 17534173 10.1097/MCP.0b013e32816b5c3b

[6] Valipour A Kreuzer A Koller H Bronchoscopy-guided topical hemostatic tamponade therapy for the management of life-threatening hemoptysis. Chest 2005;127:2113–8. 15947328 10.1378/chest.127.6.2113

[7] Cahill BC Ingbar DH Massive hemoptysis. Assessment and management. Clin Chest Med 1994;15:147–67. 8200191

[8] Sakr L Dutau H Massive hemoptysis: an update on the role of bronchoscopy in diagnosis and management. Respiration 2010;80:38–58. 20090288 10.1159/000274492

[9] De Boer WA Koolen MG Roos CM Tranexamic acid treatment of hemothorax in two patients with malignant mesothelioma. Chest 1991;100:847–8. 1889283 10.1378/chest.100.3.847

[10] Tsukamoto T H Sasaki Fau-Nakamura Nakamura H Treatment of hemoptysis patients by thrombin and fibrinogen-thrombin infusion therapy using a fiberoptic bronchoscope. Chest 1989;96:473–6. 2670463 10.1378/chest.96.3.473

[11] Bhattacharyya P Dutta A Samanta AN New procedure: bronchoscopic endobronchial sealing; a new mode of managing hemoptysis. Chest 2002;121:2066–9. 12065380 10.1378/chest.121.6.2066

[12] Lewis KM Spazierer D Urban MD Comparison of regenerated and non-regenerated oxidized cellulose hemostatic agents. Eur Surg 2013;45:213–20. 23950762 10.1007/s10353-013-0222-z PMC3739866

[13] Gabay M Boucher BA An essential primer for understanding the role of topical hemostats, surgical sealants, and adhesives for maintaining hemostasis. Pharmacotherapy 2013;33:935–55. 23686938 10.1002/phar.1291

[14] Azadani AN Mathews PB Ge L Mechanical properties of surgical glues used in aortic root replacement. Ann Thorac Surg 2009;87:1154–60. 19324142 10.1016/j.athoracsur.2008.12.072

[15] Wallace DG Cruise GM Rhee WM A tissue sealant based on reactive multifunctional polyethylene glycol. J Biomed Mater Res 2001;58:545–55. 11505430 10.1002/jbm.1053

